# Efficacy and Safety of Endoscopic Ultrasound (EUS)-Guided Lumen-Apposing Metal Stents (LAMS) as a Primary Treatment for Walled-Off Pancreatic Necrosis

**DOI:** 10.7759/cureus.78177

**Published:** 2025-01-29

**Authors:** Varun Mehta, Yogesh K Gupta, Abhinav Gupta, Yogesh Kumar, Manisha Khubber, Ajit Sood, Tanisha Sehgal, Prabhav Mehta, Ashita R Vuthaluru, Manjeet K Goyal

**Affiliations:** 1 Gastroenterology, Dayanand Medical College & Hospital, Ludhiana, IND; 2 Medicine and Surgery, Dayanand Medical College & Hospital, Ludhiana, IND; 3 Anesthesia and Critical Care, All India Institute of Medical Sciences, New Delhi, New Delhi, IND; 4 Gastroenterology and Hepatology, Dayanand Medical College & Hospital, Ludhiana, IND

**Keywords:** acute pancreatitis, endoscopy, eus, lams, minimally invasive treatment, pancreatic necrosis

## Abstract

Pancreatic necrosis, a severe complication of acute pancreatitis, is associated with significant morbidity and mortality. The use of lumen-apposing metal stents (LAMS) as a primary treatment offers a less invasive approach that may improve patient outcomes. This study evaluates the efficacy and safety of endoscopic ultrasound (EUS)-guided LAMS for treating walled-off pancreatic necrosis. In this retrospective cohort study, 95 patients treated with EUS-guided LAMS between March 2020 and October 2023 were included. Data were collected on the technical success of stent placement, clinical improvement, and management of symptomatic patients. Patients with other primary interventions, preexisting chronic pancreatitis, or incomplete clinical data were excluded. The technical success rate for LAMS placement was 100%, with a clinical success rate of 92.63%. Seven patients (7.37%) did not respond to LAMS treatment: five underwent video-assisted retroperitoneal drainage, and two had percutaneous drainage. Stent occlusion occurred in seven patients within the first week, managed through saline irrigation or direct endoscopic necrosectomy. No procedure-related complications were reported. The use of LAMS significantly reduced hospital stays and eliminated the need for additional surgeries in most cases. These findings suggest that LAMS is a highly effective and safe primary treatment for pancreatic necrosis, with high success rates and no related complications. The study’s strengths include a large sample size and comprehensive follow-up, although its retrospective, single-center design may limit generalizability. These results support the use of LAMS as a primary treatment option for pancreatic necrosis, with future research needed to refine patient selection and explore long-term outcomes.

## Introduction

Acute pancreatitis is an inflammatory process of the pancreas that can involve other local tissues or distant organ systems. It is the most common pancreatic disease. According to the revised Atlanta classification, necrotizing pancreatitis includes both pancreatic and/or peripancreatic necrosis. Approximately 45% of cases involve both pancreatic and peripancreatic tissues, while another 45% have isolated peripancreatic necrosis [1]. Pure pancreatic necrosis is seen in only about 5% of cases. Pancreatic necrosis is defined as ≥30% of non-enhancing or low-attenuating pancreatic parenchyma on a CT scan. Necrotic collections typically develop a wall after four weeks, resulting in walled-off necrosis (WON), which liquefies within five to six weeks [2].

Infected pancreatic necrosis requires drainage, whereas the indications and timing for draining sterile WON remain debated. These indications may include persistent abdominal pain, gastric outlet obstruction, or failure to thrive (characterized by ongoing systemic illness, anorexia, and weight loss) at least four weeks after the onset of acute pancreatitis. Early drainage, before four weeks, is discouraged due to the typically thick debris early in the disease, often resembling rubber [3]. After four weeks, WON can be drained surgically, endoscopically, or percutaneously, although surgical and percutaneous drainage (PCD) present significant challenges, including high complication rates and invasiveness. The decision to intervene endoscopically when the process is sterile should be carefully considered, with the management strategy depending on local expertise and the severity of comorbid conditions [4,5].

Over the past decade, endoscopic techniques have advanced significantly. The trans-papillary approach is generally inadequate for removing solid debris, whereas the transmural drainage method, which facilitates the removal of both liquefied contents and solid debris, has become the preferred approach. Initial transmural drainage techniques used trans-nasal irrigation tubes along with transmurally placed stents to lavage solid debris. Alternatively, percutaneous endoscopic gastrostomy tubes placed into the necrotic cavity helped avoid the discomfort of trans-nasal tubes. However, all these procedures had limited efficacy and were associated with numerous adverse effects and technical limitations [2,6,7].

The management of WON has been transformed by the introduction of endoscopic ultrasound (EUS)-guided lumen-apposing metal stents (LAMS). LAMS are the most commonly used stents for draining pancreatic fluid collections (PFCs) [8-10]. Previous studies have shown that LAMS are effective for all types of PFCs, with high technical and clinical success rates. Some recommend LAMS as the standard of care for WON. Direct endoscopic necrosectomy (DEN), performed by dilating the transmural tract with large-caliber balloons (up to 20 mm) or accessing a previously placed LAMS, allows for direct endoscopic access to the necrotic cavity [11,12]. Snares, grasping forceps, and other accessories are then used to remove solid debris. A retrospective study has demonstrated that this approach is superior to irrigation methods, although multiple procedures are often needed to debride residual necrotic material. Current strategies for managing WON include placing large-diameter LAMS to facilitate fluid drainage and enable DEN if needed [13].

LAMS has further revolutionized the field by enabling efficient and secure transmural drainage of fluid collections, including pancreatic necrosis. However, a comprehensive evaluation specifically focusing on the primary use of LAMS in this context is needed [14]. This study aims to systematically investigate the efficacy, safety, and outcomes associated with EUS-guided LAMS as the primary treatment for pancreatic necrosis. By building upon existing literature, this research seeks to fill a critical gap in knowledge and provide a detailed analysis of patient outcomes, procedural success rates, and potential complications. The anticipated results have the potential to shape clinical practices and guide future therapeutic strategies for patients with pancreatic necrosis.

Through this study, we aim to contribute valuable insights into the evolving field of minimally invasive techniques for treating complex pancreatic conditions. By examining patient data and procedural outcomes, this research seeks to inform evidence-based decision-making and enhance therapeutic options for clinicians managing pancreatic care.

## Materials and methods

Study design and setting

This retrospective cohort study was conducted at Dayanand Medical College & Hospital in Ludhiana, India, from March 2020 to October 2023, following approval by the institutional ethics committee (DMCH/IEC/2024/331, dated May 30, 2024). Data were systematically collected and analyzed by personnel who were blinded to the study.

Study participants

The study population consisted of adults over 18 years of age who were diagnosed with acute severe necrotizing pancreatitis, as per the revised Atlanta classification [15]. Only patients who underwent endoscopic intervention using trans-gastric LAMS as the primary treatment for WON were included. Exclusion criteria included patients under 18 years of age, those receiving primary interventions other than LAMS, individuals with preexisting chronic pancreatitis or acute interstitial pancreatitis, and those presenting with a pancreatic pseudocyst. Additionally, patients with uncorrected coagulopathy at the time of the procedure or those deemed unfit for general anesthesia by the anesthetist (blinded to the study) were excluded.

Study outline/flow

Participants who met the inclusion and exclusion criteria were enrolled in the study. Baseline demographic information, including age, gender, weight, etiology and duration of acute pancreatitis, and comorbidities, was collected. Clinical characteristics, such as presenting symptoms and their duration, severity of illness (as per the revised Atlanta classification), location, size, and necrotic component of WON, were also documented. Additionally, relevant laboratory parameters were recorded [1].

Imaging assessment

All imaging studies were reviewed by an independent radiologist, who was blinded to the clinical outcomes. The size of the necrotic collection was measured, and the presence of gas or debris was recorded. Additionally, the degree of necrosis was assessed by the endoscopist performing the procedure, as well as by a second endoscopist (using the EUS images), who was also blinded to the study. The final degree of necrosis was determined by taking the average of all three assessments.

Procedural details

**Figure 1:**
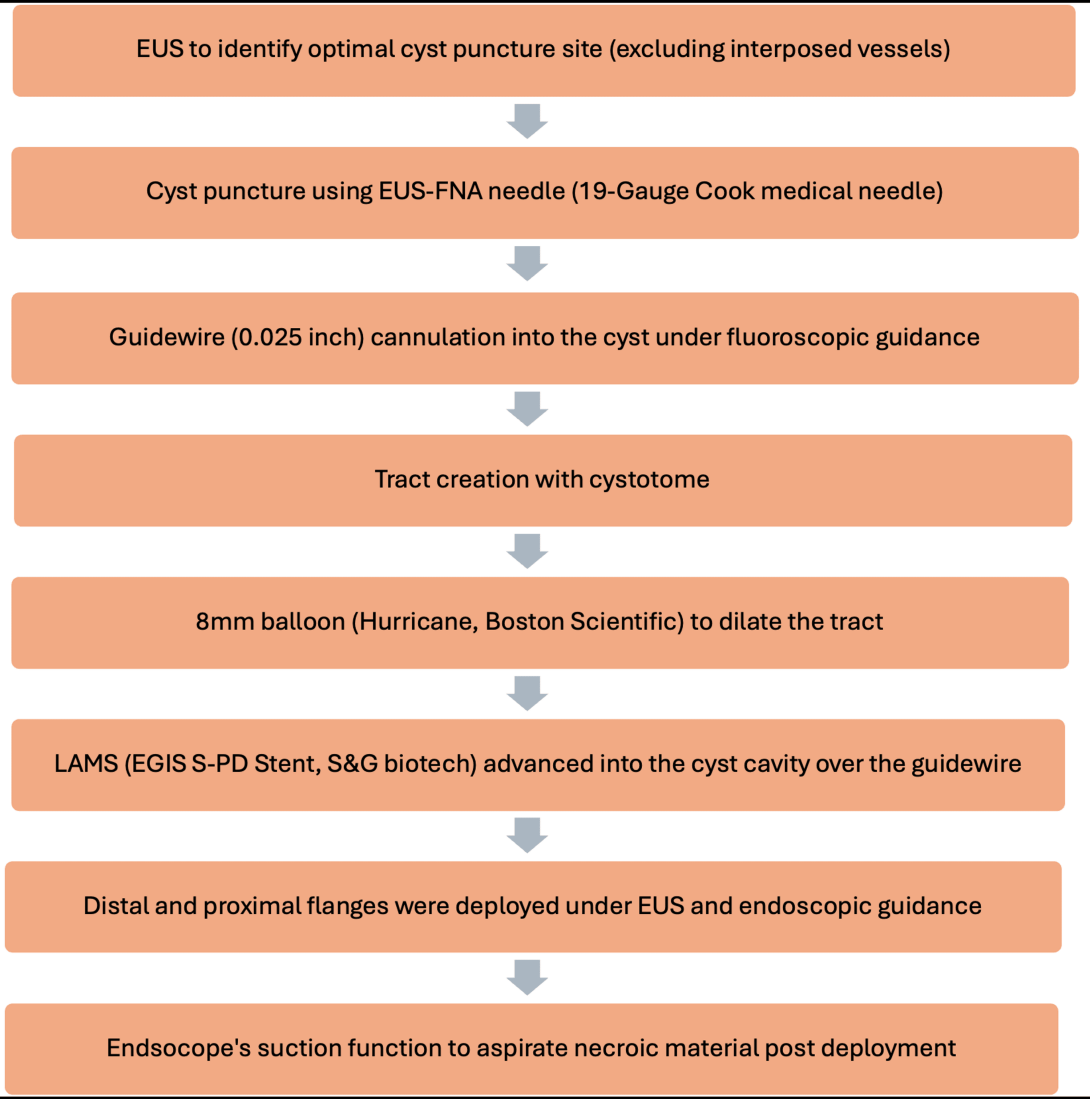
Flowchart depicting the procedural details for LAMS insertion in the drainage of WON EUS, endoscopic ultrasound; FNA, fine needle aspiration; LAMS, lumen-apposing metal stents; WON, walled-off necrosis

The LAMS (EGIS S-PD Stent, S&G Biotech, Yongin-si, South Korea) used in this study was a saddle-shaped, nitinol, braided, flexible stent fully covered with a silicon membrane (Appendix A) [16]. This fully covered, silicone-coated stent featured double flanges to secure both the stomach and duodenal wall in alignment with the WON. It was delivered via a 10F catheter with a 16 mm body diameter and a length of 30 mm, requiring a 0.035-inch guidewire for deployment (Figure 1).

Experienced endoscopists performed the procedure under general anesthesia using a therapeutic echoendoscope (Pentax EG3870UTK, PENTAX Medical, Tokyo, Japan). Broad-spectrum antibiotics were administered to reduce the risk of secondary infections. The optimal cyst puncture site (trans-gastric) was identified via EUS, with Doppler imaging ensuring no interposed vessels. A 19-gauge Cook Medical needle (Cook Medical, Bloomington, Indiana, United States) was used to puncture the WON, allowing for cyst fluid aspiration and visual inspection. A 0.025-inch guidewire was coiled into the cyst under fluoroscopic guidance, followed by tract creation with a 6 Fr cystotome. An 8-mm balloon (Hurricane™, Boston Scientific, Marlborough, Massachusetts, United States) was used to dilate the cystogastrostomy fistula before advancing the LAMS delivery catheter over the guidewire into the cyst cavity.

Under EUS and endoscopic guidance, the distal and proximal flanges were deployed sequentially, positioning the stent against the WON wall. The endoscope’s suction function was used to aspirate necrotic material after stent deployment. In cases of LAMS placement failure, alternative treatments were determined by the endoscopic and treating physician team. Empirical antibiotics were adjusted based on culture sensitivity results, following institutional guidelines.

Outcomes

The primary outcome was clinical success, defined by the resolution of WON as assessed by ultrasonography at week 12, along with clinical improvement, which was determined by the resolution of presenting symptoms. Patients requiring additional endoscopic procedures before the removal of LAMS were included in this group. The secondary outcomes included technical success (defined as the correct release of the stent at both ends, with observed drainage of the fluid), procedure time, need for additional interventions (other than endoscopic), complication rates, length of hospital stay, need for ICU care, and mortality.

Follow-up

Patient progress was meticulously documented daily during hospitalization. Patients who did not show clinical improvement within five days of LAMS insertion were managed with additional interventions at the discretion of the investigator. Stent occlusion or clogging was first addressed with saline irrigation. For cases unresponsive to saline irrigation, DEN was performed, with procedures scheduled every two to five days based on clinical response and the investigator’s judgment. Patients who did not improve with both saline irrigation and DEN were escalated to more invasive interventions, such as percutaneous drain placement and/or video-assisted retroperitoneal debridement (VARD).

After discharge, follow-up visits were scheduled biweekly for the first four weeks, during which LAMS removal was planned. Subsequent visits occurred monthly for a minimum of 48 weeks. All complications were systematically recorded and managed to ensure a comprehensive understanding of patient outcomes. Any deaths during the follow-up period were thoroughly investigated to exclude non-procedure-related causes.

Ultrasonography was the preferred imaging modality, performed at six-week intervals during the first 12 weeks and then as determined by the treating physician. To minimize radiation exposure, cross-sectional imaging was used only for specific clinical indications when ultrasonography was insufficient for decision-making.

Data sources and measurement

Data were collected using a standardized data collection form designed to capture comprehensive clinical information. The form was piloted and revised based on feedback before full-scale data extraction. To ensure accuracy and completeness, two independent researchers collected the data. Discrepancies were resolved through discussion or consultation with a third reviewer, who was blinded to the study.

Statistical methods

In this study, continuous variables were analyzed using either means with SDs or medians with IQRs, depending on the data distribution (IBM SPSS Statistics for Windows, Version 29.0 (Released 2022; IBM Corp., Armonk, NY, USA). For categorical variables, frequencies and percentages were used to summarize the data. Differences in technical and clinical success rates between groups were assessed using chi-square tests or Fisher’s exact tests, as appropriate. Continuous variables were compared between groups using either the Student’s t-test or the Mann-Whitney U test, based on the normality of data distribution. The study adhered to the STrengthening the Reporting of OBservational studies in Epidemiology (STROBE) guidelines to ensure comprehensive reporting of observational studies.

## Results

**Figure 2:**
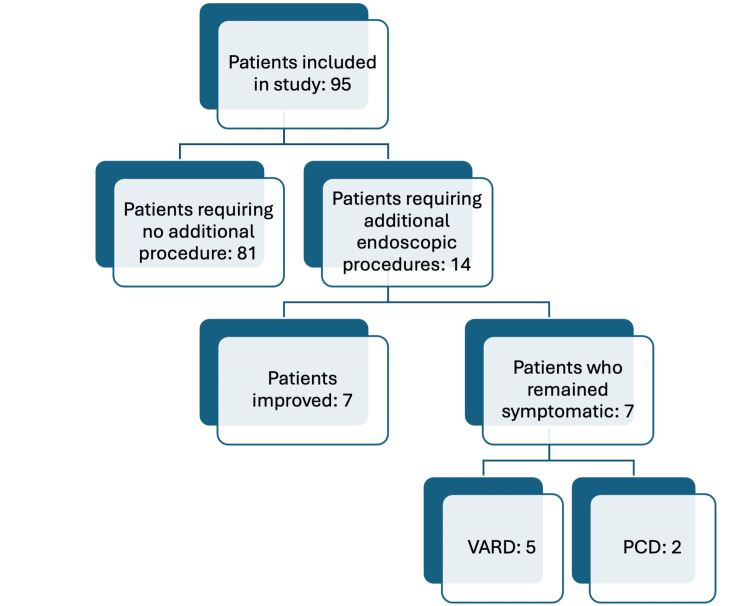
Overview of the study LAMS, lumen-apposing metal stents; PCD, percutaneous catheter drainage; VARD, video-assisted retroperitoneal debridement

A total of 95 patients were analyzed based on the inclusion and exclusion criteria. Of these, 72 (75.79%) were male and 23 (24.21%) were female. The mean age of the cohort was 35.56 ± 14.4 years, with a median age of 46 years (Table 1, Figure 2).

**Table 1:** Distribution of baseline characteristics PCT, procalcitonin; P/F, PaO2/FiO2 ratio; TLC, total leukocyte count

Characteristics	n (%)	Mean ± SD
Age (years)	-	35.56 ± 14.4
Gender (F:M)	23:72 (24.21%:75.79%)	-
Etiology		
Biliary	46 (48.42%)	-
Alcohol	38 (40%)	-
Hypercalcemia	1 (1.05%)	-
Idiopathic	10 (10.53%)	-
Severity (as per revised Atlanta classification)		
Moderately severe	61 (64.21%)	-
Severe	34 (35.79%)	-
Predominant symptom		
Pain abdomen	53 (55.79%)	
Fever with leukocytosis	32 (33.68%)	
Vomiting	10 (10.53%)	
Lab investigations		
Hemoglobin (gm/dL)	-	11.49 ± 1.68
TLC (*10³/µL)	-	10.76 ± 4.61
CRP (mg/L)	-	147.64 ± 131.5
PCT (ng/mL)	-	1.14 ± 3.65
Creatinine (mg/dL)	-	0.82 ± 0.68
Urea (mg/dL)	-	26.37 ± 25.22
Albumin (g/dL)	-	3.29 ± 0.63
P/F ratio	-	362.89 ± 55.5
Pleural effusion	40 (42.11%)	-
Respiratory rate (/min)	-	19.08 ± 4.78
Pulse rate (/min)	-	99.56 ± 13.52
Systolic blood pressure (mmHg)	-	116.06 ± 11.92

Baseline characteristics

The etiology of pancreatitis was biliary in 46 cases (48.42%), alcohol-related in 38 cases (40%), hypercalcemia in one case (1.05%), and idiopathic in 10 cases (10.53%). According to the revised Atlanta classification, 61 cases (64.21%) were classified as moderately severe, and 34 cases (35.79%) as severe [1]. The most common presenting symptoms were abdominal pain in 53 patients (55.79%), fever with leukocytosis in 32 patients (33.68%), and vomiting in 10 patients (10.53%).

Laboratory and clinical parameters

The mean hemoglobin level was 11.49 ± 1.68 g/dL, and the mean total leukocyte count was 10.76 ± 4.61 × 10³/µL. The mean CRP and procalcitonin levels were 147.64 ± 131.5 mg/L and 1.14 ± 3.65 ng/mL, respectively. Mean serum creatinine, urea, and albumin levels were 0.82 ± 0.68 mg/dL, 26.37 ± 25.22 mg/dL, and 3.29 ± 0.63 g/dL, respectively. Pleural effusion was present in 40 cases (42.11%). The respiratory rate ranged from 14 to 24 breaths per minute (mean 19.08 ± 4.78), pulse rates ranged from 88 to 126 beats per minute (mean 99.56 ± 13.52), systolic blood pressure ranged from 90 to 122 mmHg (mean 116.06 ± 11.92), and the P/F ratio had a mean of 362.89 ± 55.5.

WON characteristics and procedure details

**Figure 3:**
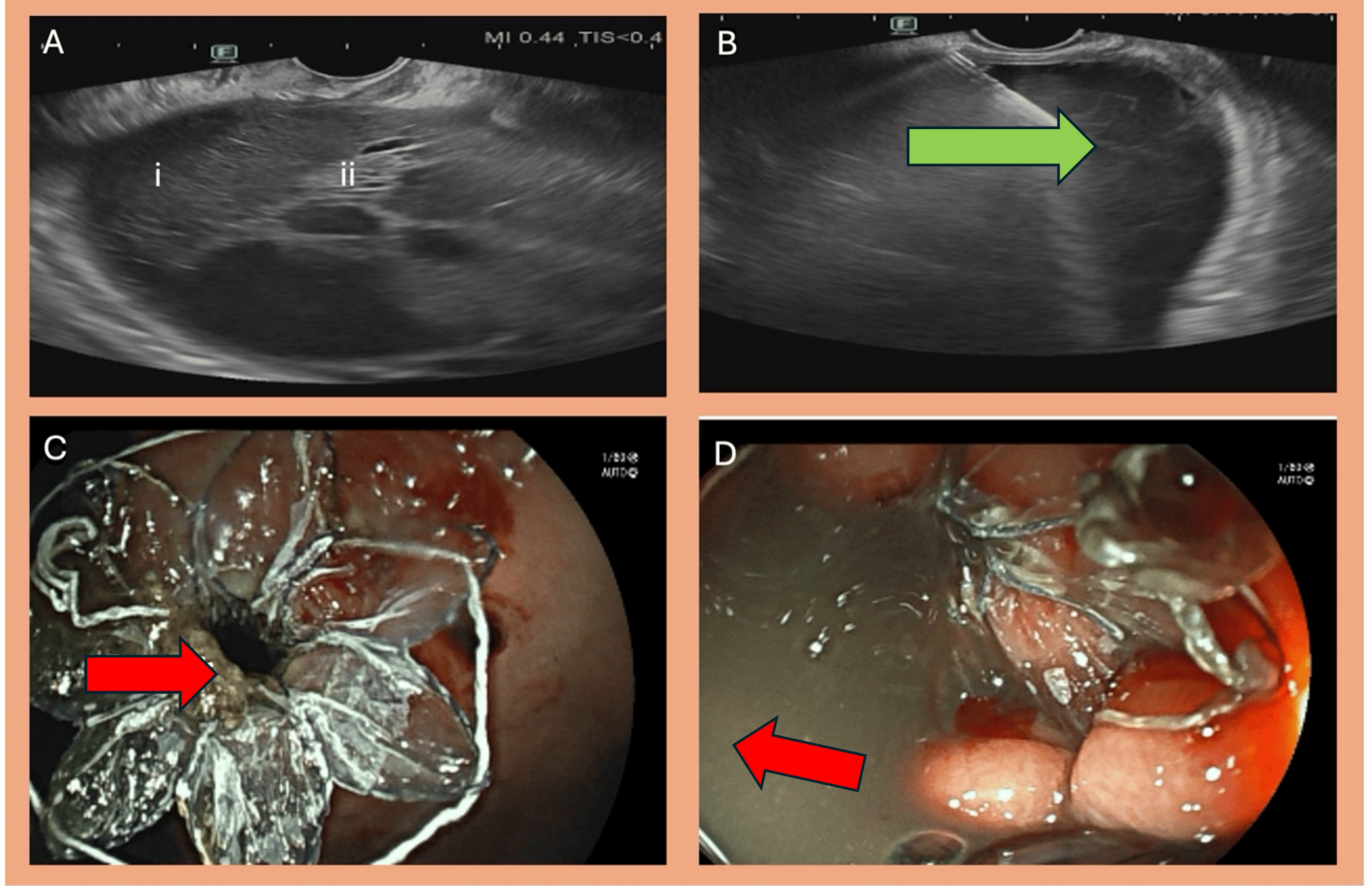
(A) EUS image in a patient with acute pancreatitis showing walled-off pancreatic necrosis, with (i) hypoechoic liquid content and (ii) hyperechoic solid content. (B) EUS image in the same patient showing FNA needle placement into the collection (green arrow). (C) Endoscopic image in the same patient showing a LAMS with solid necrotic debris efflux post-stent deployment (red arrow). (D) Endoscopic image in the same patient showing a gush of necrotic fluid (red arrow). EUS, endoscopic ultrasound; FNA, fine needle aspiration; LAMS, lumen-apposing metal stents; WON, walled-off necrosis

**Figure 4:**
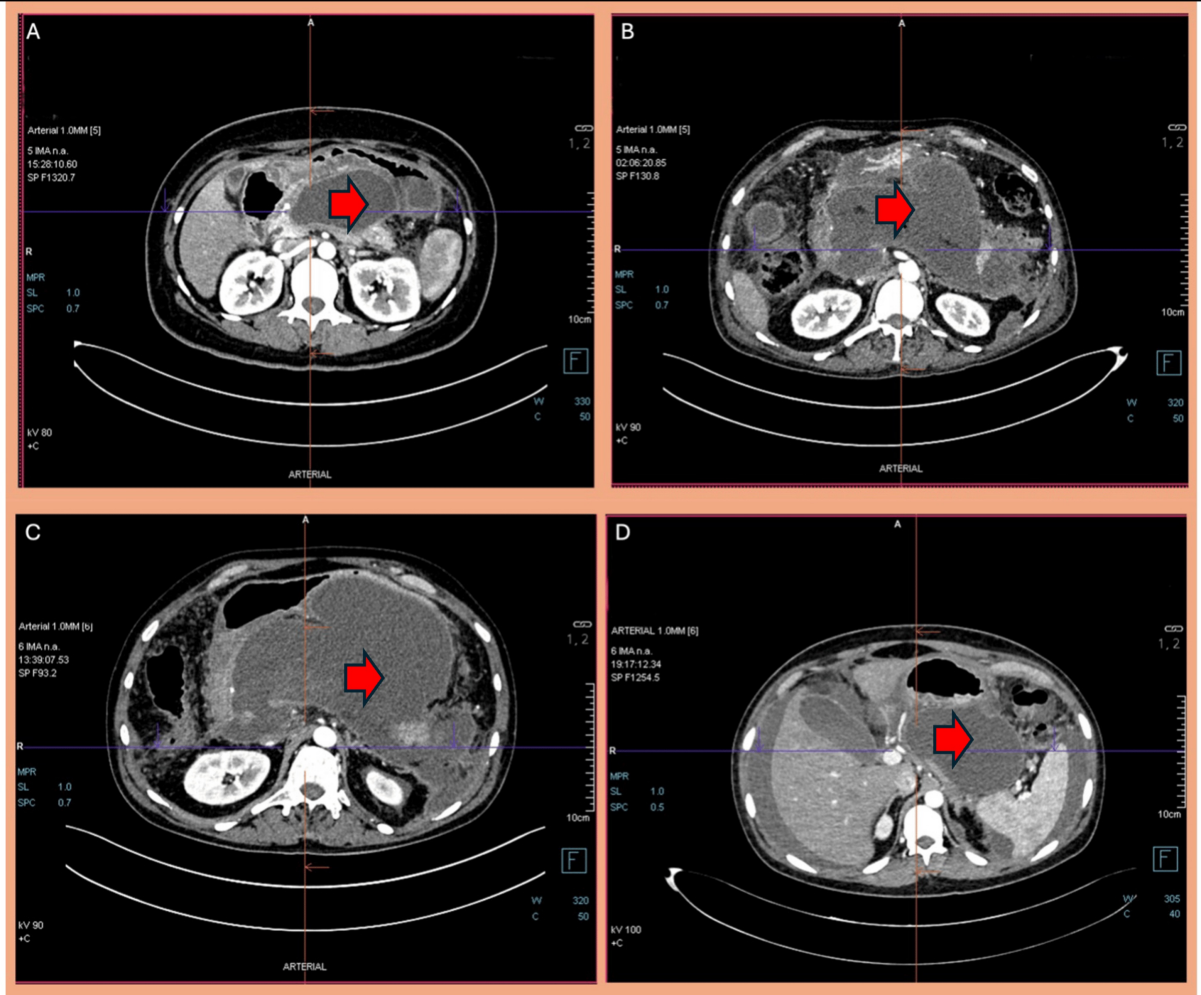
(A) CECT abdomen in a patient with acute pancreatitis showing the pancreas replaced by WON (red arrow). (B) CECT abdomen in a patient with acute pancreatitis showing a large collection replacing the bulk of the pancreas and extending into the lesser sac (red arrow). (C) CECT abdomen in a patient with acute pancreatitis showing pancreatic parenchyma replaced by a relatively well-defined collection (red arrow). The collection extends into the lesser sac, compressing and displacing the stomach anteriorly. (D) CECT abdomen in a patient with acute pancreatitis showing a well-defined collection (red arrow). CECT, contrast-enhanced CT; WON, walled-off necrosis

The mean diameter of WON was 13.8 ± 5.1 cm. The mean day of pancreatitis on which LAMS was inserted was 43.75 ± 18.03, with a median of 40 days (Table 2). The mean solid component of the necrosis was 27 ± 7% (Figure 3, Figure 4). The most common locations of WON were pancreatic and peripancreatic (42 cases, 44.21%), followed by peripancreatic alone (40 cases, 42.11%), pancreatic head (eight cases, 8.42%), and pancreatic body (five cases, 5.26%).

**Table 2:** Characteristics of WON LAMS, lumen-apposing metal stents; WON, walled-off necrosis

Parameters	n (%)	Mean ± SD
Characteristics of WON		
Day of pancreatitis on which LAMS was inserted	-	43.75 ± 18.03
Mean WON diameter (cm)	-	13.8 ± 5.1
Mean solid component of WON (%)	-	27 ± 7
Location of WON		
Head of the pancreas	8 (8.42%)	-
Body and tail of the pancreas	5 (5.26%)	-
Peripancreatic	40 (42.11%)	-
Pancreatic and peripancreatic	42 (44.21%)	-

Outcomes

Patients were discharged after a median of five days (range: three to seven days) following LAMS insertion and were followed up for a total of 48 weeks. LAMS was removed at the end of four weeks in all patients (Table 3). A total of 85 out of 95 patients did not require any additional procedures. However, 10 (10.52%) patients required further endoscopic sessions. Among these 10 patients, three (3.16%) showed improvement after additional endoscopic intervention, while two (2.11%) required PCD, and five (5.26%) underwent VARD.

**Table 3:** Outcome distribution PCD, percutaneous drainage; VARD, video-assisted retroperitoneal debridement

Parameters	n (%)
Primary outcome (i.e., clinical success at 12 weeks)	88 (92.63%)
Patients requiring no other endoscopic session	85 (88.54%)
Patients improving after additional endoscopic sessions	3 (3.16%)
Secondary outcomes	
Technical success	95 (100%)
Patients requiring additional endoscopic interventions	10 (10.52%)
Patients requiring additional interventions (other than endoscopic)	7 (7.37%)
PCD	2 (2.11%)
VARD	5 (5.26%)
Procedure time	25 ± 4.7 minutes
Median duration of hospital stays (IQR)	5 days (3, 7)
Need for ICU care	5 (5.26%)
Mortality	0 (0%)
Adverse events	
Bleeding	0
Perforation	0
Any other	0

## Discussion

This single-center retrospective study provides compelling evidence supporting the efficacy and safety of LAMS as a primary intervention for WON in patients with acute necrotizing pancreatitis. With a clinical success rate of 92.63% (88 out of 95 patients) and a 100% technical success rate, LAMS demonstrates exceptional reliability, underscoring its transformative potential in managing pancreatic necrosis. Notably, no mortality was observed in the study cohort, further reinforcing the safety profile of this minimally invasive modality.

Historically, plastic stents used for trans-gastric drainage of WON were hindered by significant limitations, including a narrower diameter and inadequate tensile strength [17]. These shortcomings often led to frequent blockages, requiring repeated interventions and increasing morbidity and mortality. In contrast, the introduction of LAMS has revolutionized the field, offering a larger diameter, enhanced tensile strength, and a saddle-shaped design that ensures superior luminal apposition and effective drainage. This innovation has fundamentally reshaped the endoscopic management of WON [18].

The results of this study align with previously reported clinical efficacy rates ranging from 73% to 98% (Table 4) [2,9,17,19-24]. However, our study uniquely extends the follow-up period to 48 weeks, providing robust evidence of LAMS’ long-term efficacy - a dimension that remains underexplored in the existing literature. By focusing exclusively on WON, this research eliminates potential biases related to data aggregation from various types of PFCs, thus offering a more precise evaluation of LAMS’ clinical utility.

**Table 4:** Recent studies on the efficacy of LAMS in the management of WON BFMS, bi-flanged metal stents; DEN, direct endoscopic necrosectomy; LAMS, lumen-apposing metal stents; Lap, laparoscopic drainage; PFC, pancreatic fluid collection; PP, pancreatic pseudocyst; PS, plastic stent; RCT, randomized controlled trial; WON, walled-off necrosis

No.	Author	Study design	Intervention	Sample size	Mean size of PFC (% solid content)	Technical efficacy	Clinical efficacy
1	Tarantino et al. (2017) [2]	Retrospective	LAMS plus DEN for WON	20	12.5 cm (-)	95%	73% (one month), 84.2% (three months)
2	Chen et al. (2019) [9]	Retrospective	LAMS vs. PS for WON	189 (102 vs. 87)	12.09 ± 5.14 cm (max >75%)	100% (LAMS), 98.9% (PS)	80.4% (LAMS), 57.5% (PS)
3	Rana et al. (2020) [17]	Retrospective	LAMS vs. PS for WON	166 (28 vs. 138)	10.8 ± 2.6 cm (30.25-40.89%)	-	98.5% (LAMS), 96.4% (PS)
4	Garg et al. (2020) [19]	RCT	LAMS vs. Lap of WON and PP	60 (30 vs. 30)	1,166.1 ± 1,086.1 cc vs. 1,355 ± 827.9 cc (max 30%)	-	90% (LAMS), 93.3% (Lap)
5	Gonsalves et al. (2023) [20]	RCT	LAMS vs. PS for WON	61 (30 vs. 31)	-	-	63% (LAMS), 45% (PS)
6	Siddiqui et al. (2021) [21]	Retrospective	BFMS vs. LAMS plus DEN in WON	387 (205 vs. 182)	11.29 cm (-)	99% in each	96.1% (BFMS), 95.6% (LAMS and DEN)
7	Yang et al. (2018) [22]	Retrospective	LAMS for WON and PP	122 (64 vs. 58)	10.6 cm (-)	-	62.3% (WON), 96.5% (PP)
8	Maharshi et al. (2021) [23]	RCT	Nasocystic irrigation with H_2_O_2_ vs. LAMS in WON	50 (25 vs. 25)	10.3 ± 4.1 cm (32 ± 10.4%)	100% (H_2_O_2_), 96% (LAMS)	83.33% (H_2_O_2_), 78.3% (LAMS)
9	Angadi et al. (2021) [24]	RCT	Endoscopic (LAMS/PS) vs. Lap of WON	40 (20 vs. 20)	1,586.5 ± 505.2 ml vs. 1,229.4 ± 751.2 ml (max >1/3)	-	Four weeks: 80% (Lap), 75% (LAMS and PS); Overall: 90% (Lap), 85% (LAMS and PS)

Several factors contributed to the high success rates observed in this study. On-site drainage, performed during stent placement and averaging 800-1500 mL, was crucial in expediting symptom resolution and reducing the risk of sepsis by promptly decompressing infected fluid collections. Additionally, targeted antibiotic therapy based on fluid aspirate cultures, precise EUS-guided puncture near the gastric wall, and oblique stent deployment were key in enhancing drainage efficiency and minimizing complications. These measures collectively reduced hospital stays to an average of five to six days and eliminated the need for ICU admissions.

Although this study is retrospective, it represents one of the largest single-center series from India, lending considerable weight to its conclusions. Our study also emphasizes the role of precise EUS-guided techniques, which integrated endoscopic, fluoroscopic, and EUS guidance to achieve effective drainage while minimizing procedural complications. This comprehensive approach highlights the importance of advanced imaging modalities in refining stent deployment and maximizing therapeutic outcomes.

Despite its strengths, the study has limitations, underscoring the need for prospective, multicenter trials to validate these findings and establish standardized protocols for LAMS deployment. Such efforts will be essential in defining optimal patient selection criteria, refining procedural techniques, and assessing cost-effectiveness, ultimately facilitating the broader adoption of LAMS in clinical practice.

## Conclusions

This study unequivocally demonstrates that EUS-guided drainage of WON using LAMS is a safe, effective, and durable treatment option. On-site drainage during stent placement not only accelerates clinical improvement but also mitigates the risk of sepsis and reduces procedural complications, emphasizing its critical role in optimizing LAMS deployment. By simplifying the management of WON and minimizing procedural morbidity, LAMS provides a minimally invasive alternative to traditional surgical methods. With its exceptional efficacy and safety profile, LAMS is poised to become a cornerstone in the treatment of WON. Future prospective, multicenter trials are crucial to further validate these findings and establish LAMS as the gold standard for WON management in clinical practice.

## Figures and Tables

**Figure 5:**
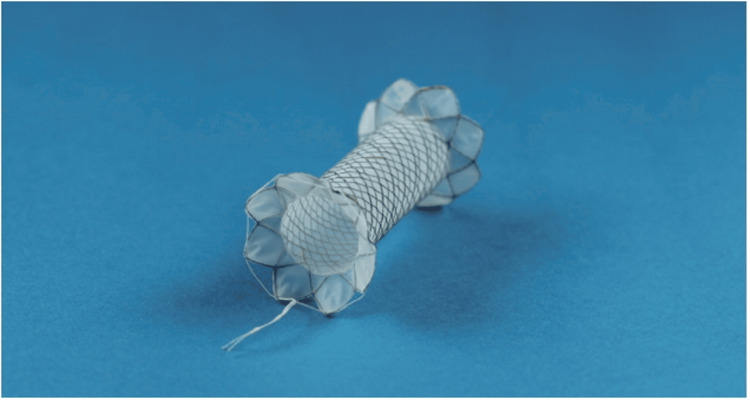
Photograph of EGIS LAMS stent used in the study LAMS, lumen-apposing metal stents

## Appendices

Appendix A

Appendix B

**Table 5:** Specifications of various LAMS used worldwide LAMS, lumen-apposing metal stents

Manufacturer	Model	Internal diameter (mm)	Length (mm)	Flange diameter (mm)	Material composition
Boston Scientific	AXIOS	10, 15	10	21, 24	Nitinol wire, fully covered with a silicone membrane
Taewoong Medical	SPAXUS	8, 10, 16	20	25	Nitinol wire, fully covered with a silicone membrane
Taewoong Medical	Nagi	10, 12, 14, 16	10, 20, 30	22, 24, 26, 28	Nitinol wire, fully covered with a silicone membrane
S&G Biotech, Korea	EGIS S-PD stent	10	20, 30, 40	20	Nitinol wire, fully covered with a silicone membrane
